# Development of a community-based COVID-19 intervention in rural Ghana: a document analysis

**DOI:** 10.1186/s12889-022-14338-8

**Published:** 2022-10-14

**Authors:** Shadrack Osei Frimpong, Moro Seidu, Sam Kris Hilton, Yusuf Ransome, Elijah Paintsil, Kristina Talbert-Slagle, Sharon Dorcoo-Attipoe, Carol Brayne

**Affiliations:** 1 Cocoa360, Accra, Ghana; 2 Department of Public Health and Primary Care, University of Cambridge, Cambridge, England; 3 Department of Social and Behavioural Science, Yale School of Public Health, Yale University, New Haven, USA; 4 Department of Pediatrics, Yale School of Medicine, Yale University, New Haven, USA; 5 Departmemt of Epidemiology of Microbial Diseases, Yale School of Public Health, Yale University, New Haven, USA; 6 TERSHA LLC, Alpharetta, GA USA

**Keywords:** COVID-19, Cocoa360; CoCoPOPP, PARIHS, Rural Ghana

## Abstract

**Background:**

The COVID-19 pandemic has caused the loss of millions of lives and economic breakdowns in many countries across the globe. Despite the limited availability of vaccines and the challenges of poor health infrastructure, few interventions have been developed and implemented for those who live in rural areas, particularly in sub-Saharan Africa. In response, Cocoa360, a global health nonprofit in rural Ghana designed an intervention called Cocoa360’s COVID-19 Preparedness and Outbreak Prevention Plan (CoCoPOPP). This paper aimed to examine the extent to which CoCoPOPP’s design aligned with the Promoting Action on Research Implementation in Health Services (PARIHS) framework.

**Methods:**

We reviewed documents influencing CoCoPOPP’s design between March and June 2021. A total of 11 documents were identified for analysis. Using the Promoting Action on Research Implementation in Health Services (PARIHS) framework as a guide, thematic analysis was done to analyze the extracted data.

**Results:**

Overall, CoCoPOPP’s design aligned with the evidence, context, and facilitation domains of the PARIHS framework. It positioned CoCoPOPP as an intervention that considered the unique context of a rural Ghanaian setting. It was guided by robust and high-quality published and non-published evidence and engaged external and internal stakeholders during its implementation. CoCoPOPP’s context-dependent nature positions it for potential replication in sub-Saharan Africa’s rural communities with similar farming contexts. Specific areas that were less well and/or not addressed were the unintended negative consequences of community engagement, the absence of primary data in the guiding evidence, and the lack of a facilitation continuum coupled with the role of power during the facilitation process.

**Conclusion:**

CoCoPOPP, Cocoa360’s response to the COVID-19 pandemic in rural Ghana, is an evidence-driven, context-dependent public health intervention that has been designed to reduce COVID-19 infections and prevent potential deaths. This study underscores the importance of considering the unique community and cultural contexts, employing evidence, and engaging local and external actors as facilitators when designing interventions to respond to global health pandemics.

## Introduction

The coronavirus disease 2019 (COVID-19) has thrown the entire world into disarray – hundreds of thousands of lives have been lost, economies have come to a halt, and the urgency to stop its spread has grown daily. In March 2021, over 123 million COVID-19 cases had been confirmed worldwide, of whom 69.9 million people have recovered, and 2.7 million have died [1]. Although the USA, India and Brazil have been the most affected countries, low-and-middle-income countries in sub-Saharan Africa continue to experience surges in infections. Notably, the continent has recorded over 4 million confirmed cases (with South Africa being the most drastically affected country), 3.57 million recoveries, and an estimated 106,280 deaths [2]. Ghana, one of the African nations with a relatively high incidence of COVID-19 (in the top 10), has confirmed nearly 90,000 cases, 86,000 recoveries, and a little over 700 deaths as of mid-March 2021 [3]. Yet, with under a million people tested out of the country’s 30 million population, these numbers are just a small percentage [3].

Evidence on actual COVID-19 occurrence in the populations of Ghana and other African countries will be highly underestimated, and existing civic systems cannot capture the scale of community transmissions, particularly in rural areas [4]. Rural communities in developing countries are considerably more vulnerable to COVID-19 than urban areas [5, 6] because the population is relatively older [7]. Economic pressures, out-migration of the young and return of retirees have all been shown to contribute to this ageing of rural communities [7–10]. Coupled with the high prevalence of comorbidities such as diabetes, cardiovascular diseases, and lung diseases, these economic and migratory pressures exacerbate the potential impact of the pandemic in these communities [11, 12]. The healthcare challenges posed by the COVID-19 pandemic are a significant concern for rural areas, where over 70% of Ghana’s rural population already struggle to access healthcare [4].

Given the diversity of demographic, socio-cultural and economic circumstances across nations of the world, and those recommendations from the World Health Organisation (WHO) are framed at a global level, each country and locality must contextualize and adapt WHO’s recommendations [13]. The reality of such contextual differences has resulted in in-country innovations and adaptations to the pandemic response, including local solutions such as mobile-driven self-diagnosis applications, an X-ray-based self-screening platform, mobile-based screening and mapping tools, low-cost methods for the production of personal protective equipment (PPE) being implemented across sub-Saharan Africa [13]. Such innovation, driven by urgent need and mostly without in-built evaluation, highlights how important it is to generate evidence on effective community-specific interventions to control the spread of COVID-19 and alleviate its impact on health and socio-economic conditions for rural citizens. In Ghana, the government has provided scant support to rural communities [4]. During the COVID-19 pandemic, this situation has not changed, and the COVID-19 prevention and control efforts in most rural areas in Ghana and other developing countries have fallen short of in their capacity to improve health outcomes [5, 6, 14, 15].

**Fig. 1:**
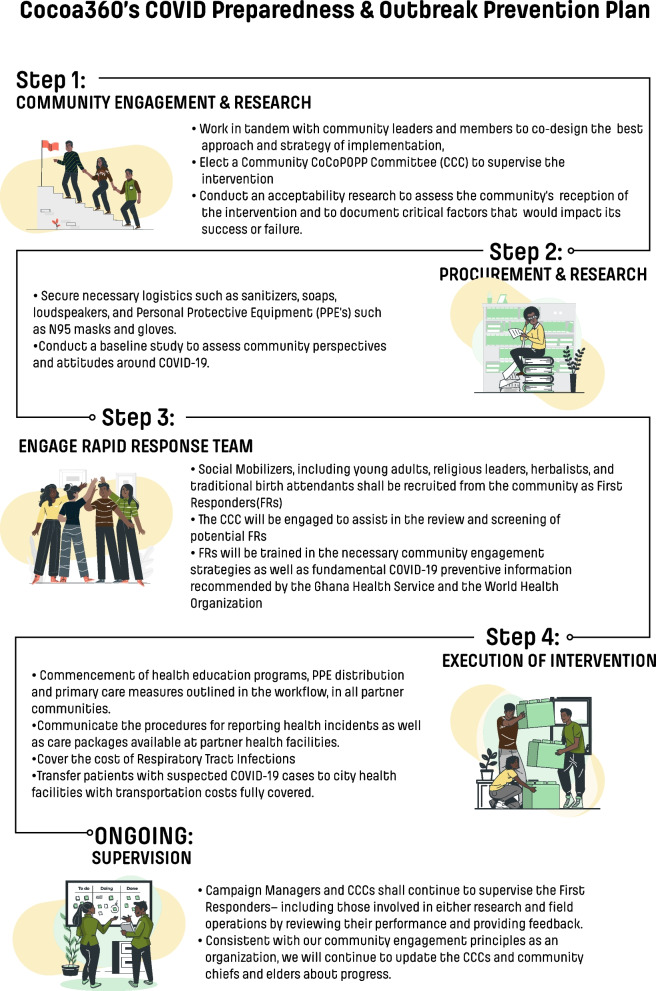
Schematic of Cocoa360's COVID Preparedness and Outbreak Prevention Plan (CoCoPOPP)

In many rural Ghanaian communities, few COVID-19 interventions have been tailored to their unique cultural and socio-demographic needs [4]. In rural Western Ghana, a not-for-profit organization (Cocoa360) was already in place, leveraging community engagement to address healthcare and educational access challenges. Cocoa360 was well-positioned to facilitate one of the rural responses to the COVID-19 pandemic [16, 17]. The organization rapidly developed and implemented a collaborative intervention called COVID-19 Preparedness and Outbreak Prevention Plan (CoCoPOPP) in the eight rural, remote communities it serves (Fig. 1). These communities’ unique rural and remote locations allow research on pandemic management and control, which can later be scaled to other rural areas. CoCoPOPP was designed to ensure that rural inhabitants are educated about COVID-19, and access PPE and high-quality healthcare services by eliminating treatment fees for respiratory tract infection cases at Cocoa360’s clinic.

Given the paucity of evidence on how public health interventions are designed in response to global pandemics, this paper employs document review and thematic analysis using the Promoting Action on Research Implementation in Health Services (PARIHS) framework. Our goal is to examine the extent to which CoCoPOPP’s design aligns with the components of the PARIHS framework. In this paper, we reviewed documents that informed CoCoPOPP’s design and used the PARIHS framework to thematically analyze the data we extracted. Such review and analysis are crucial for the future scaling of CoCoPOPP in rural communities with similar contexts. In addition, we share learnings from this process for development professionals in rural areas who seek to scale up participatory knowledge translation research and facilitate engagement at the community level.

## Method

### Document review and the PARIHS framework

As a qualitative research method, document analysis can serve as an essential research tool either as a stand-alone or as part of a triangulation scheme [18]. It is mainly used as a stand-alone methodology when in-person approaches such as participant observations, interviews, and questionnaires are restricted by health and distance concerns [19]. Given the limitations in communication and travelling during the COVID-19 pandemic, the authors employed a methodology comprising both document review and thematic analysis using a public health framework. In addition to preventing health risks, document analysis proved a more suitable approach to circumvent potential challenges of distrust, hostility and retaliation toward researchers that typically happen during global pandemics [20–22]. Specifically, this study relied on Dalglish et al.’s approach to document review in health policy research: the READ methodology [23]. The READ approach comprises 1) Readying the materials under investigation, 2) Extracting data from the materials, 3) Analyzing the data, and 4) Distilling the findings from the data.

The Promoting Action on Research Implementation in Health Services (PARIHS) framework was employed for rigorous data analysis and distillation. PARIHS was developed to help professionals implement research into practice [24]. It structures the capture of evidence to use at the implementation level and considers its broader implementation context [25, 26]. At PARIHS’ the core are three key elements: level and nature of evidence, the context in which the research is to be applied, and facilitation of the implementation process [24]. With a strong emphasis on these three key elements, the framework provides essential guidelines for ensuring that interventions achieve the highest favourable outcomes with minimal unintended negative consequences when implemented. Several empirical studies corroborate the PARIHS framework’s strength by demonstrating that successful implementation is a function of evidence, context, and facilitation [24, 27–31]. The most successful implementation occurs when: the evidence is scientifically robust and matches professional consensus and target population needs (‘high’ evidence); the context is receptive to change with sympathetic cultures, strong leadership, and appropriate monitoring and feedback systems (‘high’ context); and there is appropriate facilitation of change with input from skilled external and internal facilitators (‘high’ facilitation) [24, 32].

### Data collection and analysis

Combining the READ approach and the PARIHS framework analysis, this study rigorously reviewed and analyzed key documents that applied to developing the CoCoPOPP intervention as below.

#### Step 1: readying materials

Eleven documents related to CoCoPOPP’s development between 2019 and 2022 were obtained from Cocoa360’s staff, executives, research partners, and the internet. Records included the initial document outlining CoCoPOPP’s implementation plan, a logic model describing key inputs and outputs of the intervention, promotional materials such as brochures and videos, CoCoPOPP’s operational flowchart, donor, and partner update reports, as well as related press and articles on the internet. Document acquisition was greatly facilitated by Cocoa360’s executives and board members, who suggested additional documents such as grant reports, typically deemed confidential and inaccessible. All authors worked together to ensure that the final documents selected for the review adhered to Flick’s four yardsticks of document selection: authenticity, credibility, representativeness, and meaning [33].

#### Step 2: extracting the data

Data from the documents and internet-based searches were then extracted into a Microsoft Excel file, structuring the information according to the name/title, year of publication, source, aims and objectives of the document. Table 1 outlines the 11 documents that were reviewed and the key messages that were extracted.

**Table 1:** List of documents identified and analyzed

Name/Title	Year Published	Aims and Objectives	Source
CoCoPOPP implementation document	2020	To explain the rationale and processes for effective CoCoPOPP implementation	Cocoa360 (Available upon request)
CoCoPoPP Logic Model	2021	To provide a blueprint for CoCoPOPP’s replication in rural communities with similar contexts.	Cocoa360 (Available upon request)
A Case for Girl-child Education to Prevent and Curb the Impact of Emerging Infectious Diseases Epidemics	2020	To review the available evidence of sustainable educational models and their impact on health systems	https://pubmed.ncbi.nlm.nih.gov/33005122/
CoCoPOPP brochure	2020	Health communication material for CoCoPOPP	Cocoa360 (Available upon request)
CoCoPoPP Video	2020	Promotional material for CoCoPOPP	https://www.youtube.com/watch?v=FMVZ9G7RqWM
Grant Report for the Clinton Global Initiative	2021	To update the Clinton Foundation on a grant they provided towards CoCoPoPP’s implementation	Cocoa360 (Available upon request)
Grant Report for the Queen’s Commonwealth Trust	2021	To update the Queen’s Commonwealth Trust on a grant they provided towards CoCoPoPP’s implementation	Cocoa360 (Available upon request)
Grant Report for Yale Global Health Institute	2021	To update Yale Global Health Institute on a grant they provided towards CoCoPoPP’s implementation	Cocoa360 (Available upon request)
Cocoa360’s innovative means of effectively battling the COVID-19 pandemic in rural areas	2021	Highlighting the impact of CoCoPOPP in the targeted rural communities	https://thebftonline.com/2021/03/03/cocoa360s-innovative-means-effectively-battling-covid-19-pandemic-in-rural-areas/
Rural Zero Campaign Presentation	2021	Presentation materials on CoCoPOPP for Cocoa360’s donors and partners	Cocoa360 (Available upon request)
Cocoa360’s 2020 Annual Report	2021	To share Cocoa360’s impact and progress of work in the previous year	https://cocoa360.org/wp-content/uploads/2022/06/2022-Annual-Report.pdf

#### Step 3: analyzing the data

Relevant information from the extracted was organized according to the different components of the PARIHS framework. We specifically applied the evidence, context, and facilitation elements of the PARIHS framework in designing the CoCoPOPP intervention. These elements interact in robust and complex ways to influence CoCoPOPP’s implementation effectiveness. Two authors (SOF and MS) also conducted reflexive journaling to document how their pre-standing views and characteristics as Cocoa360 executives might have influenced the findings from the data analysis [34]. Finally, authors who are not Cocoa360 staff, including CB, EP, SA-D and YR, rated the CoCoPOPP intervention against the individual components of the PARIHS framework to minimize bias and improve rigour (Table 2).

**Table 2:** CoCoPOPP satisfying PARIHS framework elements and sub-elements

Elements	Sub-element	Rating
**Evidence**	Research	High
	Professional (Clinical) Experience	Moderate
	Community preference	High
**Context**	Leadership	High
	Culture	High
	Evaluation (Measurement)	High
**Facilitation**	Characteristics of facilitator	High
	Role of facilitator	High
	Style of facilitator	High

#### Step 4: distilling the findings

Using Dalglish et al.’s measures of data distillation, document analysis was determined to be wholly based on data saturation; the authors have read enough documents to be sufficiently confident about how Cocoa360 designed the CoCoPOPP intervention [23]. Data from framework analysis of the CoCoPOPP intervention was then distilled into the different components of the PARIHS framework, namely, evidence (research, professional experience, and community preference), context (culture, leadership, and evaluation), and facilitation (characteristics, role and style of the facilitators) (Table 3).

**Table 3:** Summary of how CoCoPOPP’s design aligned with the PARIHS Framework

**Evidence**		
**Sub-domains**	**PARIHS definition**	**CoCoPOPP application**
Scientific Research	Evidence needs to be translated and adapted to make sense in the local context. Research evidence is less specific and less value-free than is often acknowledged.	The intervention’s design was scientifically robust as it relied on the research of published sources and matched professional opinions reached by the design group.
Clinical Experience	The tacit knowledge of practitioners, or ‘practical know-how’, must be explicit for practitioner expertise to be shared, critiqued, and developed.	CoCoPOPP’s design team relied on the expert opinions and experiences of professionals. Physicians and clinical practitioners from GHS—PHVHD, TBCC, and TBCHPS—who understand the socio-cultural dynamics, disease prevalence, demographics, health care needs, and services utilization of the communities were part of the design team.
Patient Experience	Inclusion of groups and communities in decision-making.	The intervention’s design included the formation of a COVID-19 committee comprising community leaders and members who can make and influence decisions concerning its implementation.
Local Information	Data on the local context, such as evaluation data, local community stories and knowledge of the organizational culture, must be considered.	The design team considered community perspectives and routine information derived from the members of the communities.
**Context**		
**Sub-domains**	**PARIHS definition**	**CoCoPOPP application**
Culture	Intervention is designed to meet the cultural dynamics of the communities	During CoCoPOPP’s design, the design team gave a significant mandate to the Chief and elders (who are the custodians of the communities) by seeking their approval before the intervention was unveiled for implementation.
Leadership Roles	Clear roles and objectives among stakeholders	Clearly defined roles, responsibilities, objectives, and effective coordination specified for each of the stakeholders and among the various team units
Evaluation	Robust monitoring and evaluation structures	Interdisciplinary investigators from Yale University*,* Vanderbilt University, University of Ghana, MoH, GHS, and Cocoa360 were noted as participants in CoCoPOPP’s monitoring and evaluation efforts. The intervention package further allowed for data collection before, during, and after implementation to measure the effectiveness of all possible activities and outcomes. The intervention design also factored in all the necessary metrics to estimate the possible individual and team performance, activities, outputs, outcomes, and impact of the intervention.
**Facilitation**		
**Sub-domains**	**PARIHS definition**	**CoCoPOPP application**
Characteristics of facilitators	openness, credible, authentic	The village chief and elders are the opinion leaders of the local communities. The communities recognize as highly credible, respected sources of influence (via authority, status, and representativeness).
Roles of facilitators	clarity of role, authority	To ensure consistency, the facilitators had clearly defined roles to achieve a specific objective in CoCoPOPP’s design and implementation.
Facilitation style	range and flexibility of style, consistent and appropriate presence	Facilitators, especially those directly involved in the intervention’s success, had the experience of at least two years in the environment of the intervention area and were fully aware of the possible challenges they were likely to face. Hence, they were flexible, showed empathy when dealing with people, and were tenacious in overcoming challenges.

## Results

### Satisfying PARIHS’ evidence in the design of CoCoPOPP

CoCoPOPP’s design was evidence-informed because it relied on the research of published sources, matched professional opinion reached by the group, and met the needs of communities involved in the CoCoPOPP intervention. The design process considered the needs of the target communities because it depended on community perspectives and routine information derived from the members of the communities.

The design team also relied on the expert opinions and experiences of professionals. Physicians and clinical practitioners from the Ghana Health Service — Prestea-Huni Valley Municipal District (GHS-PHVMD), Tarkwa Breman Community Clinic (TBCC), and the Tarkwa Breman Community Health Planning Services (TBCHPS) — who understand the socio-cultural dynamics, disease prevalence, demographics, health care needs, and services utilization of the communities, were included. While the design team acknowledges the constraints of time and resources due to the COVID-19 pandemic, they also conducted an umbrella review of the literature regarding the design, implementation, and outcomes of previous pandemics such as the Ebola Virus Disease (Table 4).

**Table 4:** List of articles on pandemics reviewed for CoCoPOPP^28^

Article	Meth Limit.	Relevance	Coherence	Adeq. of Data	OCAoF
Frimpong & Paintsil (2020); Ebola [48]	MC	MC	MC	MC	HC
Coltart et al. (2017)- Ebola [49]	MC	MC	MC	MC	MC
Kirsch et al. (2017); Ebola [22]	MC	MC	MC	MC	MC
Cornish et al. (2014); HIV/AIDS [50]	MC	MC	MC	MC	MC
Salam et al. (2014); HIV/AIDS [51]	MC	MC	MC	MC	MC
McLean et al. (2018); Ebola [52]	MC	MC	MC	MC	HC
Abramowitz et al. (2015, 2017); Ebola [53, 54]	MC	MC	MC	MC	HC
Sambala et al. (2019); Influenza [55]	MC	MC	MC	MC	HC
WHO Ebola Response Team (2018) [56]	MC	MC	MC	MC	HC

*Meth Limit* Methodological limitations, *Adeq. of Data* Adequacy of Data, *OCAoF* Overall CERQUAL Assessment of Confidence, *MC* Minor Concerns, *HC* High confidence

Source: Compiled from CoCoPOPP implementation document, 2020

### Satisfying PARIHS’ context in the design of CoCoPOPP

The design of CoCoPOPP matched the needs of the target population. The team considered the communities’ culture while acknowledging and including the participating rural communities’ leadership, monitoring, and feedback systems.

#### Culture context of CoCoPOPP

The intervention was designed to meet the cultural dynamics of the communities. As part of the planned implementation strategy, it was specified that:

CoCoPOPP will first be presented to the Chief and elders of Tarkwa Breman (TB) for feedback, support, and suggestions. Also, request that a community leader (preferably the local Chief) announce CoCoPOPP to the community, highlighting the community’s risk and the intervention’s potential impact and encouraging interested residents to sign up for social mobilization roles [35].

This planned implementation strategy gave a more significant mandate to the Chief and elders (who are the community custodians) to approve the intervention before it was unveiled for implementation. Hence, the following was documented in the design of the implementation strategy:

After approval from community leaders and Cocoa360’s Village Committee (VC), we shall secure the necessary logistics [35].

Also, the intervention was designed to ensure that the community leads and champions the communication aspect of the intervention.

Request community leaders to champion CoCoPOPP: Take the lead in telling the community about CoCoPOPP and cultivating their support [35].

Moreover, the design of the intervention-implementation strategy also ensured that the community members did not only benefit from the intervention but also took active roles in the implementation process and were treated as experts [*see excerpts from the intervention document below*].

Requesting community leaders (preferably the local Chief and VC) to encourage interested residents to sign up for social mobilization roles …; All participants recruited for the surveys and focus group discussion are treated as experts [35].

The study ensured that all participants were respected and treated as experts, reimbursed their travelling costs (if any), and received souvenirs (such as prepaid phone cards after interview /focus discussions). Instead of cash, gifts were provided to the participants considering their communities’ context and cultural norms. The plan also recognized potential acceptability, trust, recognition, and respect issues. It recommended ways to minimize retaliation by engaging the community leaders and VC in introducing CoCoPOPP to the communities. Furthermore, the recruitment announcement for CoCoPOPP’s research assistants was first delivered by local leaders at a community meeting. Similarly, community leaders were included in the discussions to promote community members’ participation.

CoCoPOPP’s design plan also included educating the population, promoting learning in the communities, and conducting research to collect data to try new and different techniques for organizational use. Further, the plan focused on sharing insights on epidemic management and control with the Ghanaian government and the wider global health and education community [*see excerpts from the intervention document below*].

The intervention presents a strong opportunity to research and gain insights on epidemic management and control with the Ghanaian government and the broader global health and education community. This will be crucial for controlling and managing future epidemics in similar settings [35].

The design team also noted that these implementation measures would increase CoCoPOPP’s likelihood of success in minimizing the spread of COVID-19 in the community while following the cultural dynamics of the local people.

#### Leadership context of CoCoPOPP

CoCoPOPP’s design outlined clear roles and objectives among the stakeholders involved in the intervention. It notes that stakeholders within each group must work together as a team and share power. For instance, TBCC healthcare workers worked closely with each other and had general authority in treating their clients. Each micro team, including clinical staff, and research assistants, was coordinated by the Cocoa360 managers to ensure harmony and good communication among the teams. A high sense of leadership characterized CoCoPOPP’s design because of the clearly defined roles, responsibilities, objectives, and effective coordination specified for each stakeholder and among the various team units (Table 5).

**Table 5:** Stakeholders involved in the implementation of CoCoPOPP

Primary stakeholders	Secondary Stakeholders
Community leaders (Chief and elders, Village Committee (VC) Community members Cocoa360 executives and directors TBCC healthcare workers Cocoa360 Research Team Social mobilizers Community liaison Information flow manager Data collectors TBCHPS	University of Ghana Ministry of Health (MoH) Vanderbilt University Yale University Donors

Source: Compiled from CoCoPOPP implementation document, 2020

#### Evaluation of CoCoPOPP

Evaluation is one of the critical fulcrums CoCoPOPP’s design team leveraged. The intervention strategy allowed interdisciplinary investigators from Yale University*,* Vanderbilt University, University of Ghana, Ministry of Health (MoH), Ghana’s Health Service (GHS), and Cocoa360 to participate in monitoring and evaluation activities. Below is an excerpt from the implementation strategy, highlighting how CoCoPOPP was consistent with the PARIHS framework’s sub-element evaluation.

A strong team of interdisciplinary investigators at the University of Ghana and Yale University, in partnership with (MoH) (GHS), Cocoa360, and VC, shall research to monitor and evaluate the CoCoPOPP intervention [35].

The intervention package further allowed for data collection before, during, and after implementation to measure the effectiveness of all possible activities and outcomes. Likewise, the intervention design also factored in all the necessary metrics to estimate the possible individual and team performance, activities, outputs, outcomes, and impact of the intervention. CoCoPOPP’s design also emphasized feedback on individuals, the team, and the intervention performance in the community.

Consistent with our community engagement principles as an organization, we will continue to update VC, community chiefs, and elders about progress → materials distributed; cases being seen [35].

### Satisfying PARIHS facilitation in the design of CoCoPOPP

Facilitation is an element in the PARIHS framework, a function of implementation success and is influential in overcoming the barriers to evidence-based practice [36]. The designers of CoCoPOPP took facilitation into account in the design process by soliciting inputs from relevant internal and external facilitators. Internal facilitators include community leaders (Chief and elders, VC), Cocoa360 executives, TBCC healthcare workers, social mobilizers, Cocoa360’s research team, and data collectors. In contrast, the external facilitators were representatives from Yale University, Vanderbilt University, the University of Ghana, and MoH. These facilitators exhibited characteristics consistent with opinion leaders, change agents, champions, educational outreach workers, and linking agents in the implementation strategy to promote high facilitation.

With regards to community facilitation of CoCoPOPP, the Chief and elders were noted as the opinion leaders from the local communities. The communities view them as highly credible, respected sources of influence (via authority, status, and representativeness). The VC helped coordinate implementation synergy between Cocoa360 and members of the participating communities. Lastly, Cocoa360’s executives, TBCC healthcare workers, Cocoa360’s research team, social mobilizers, and data collectors were the internal change agents who promoted and ensured CoCoPOPP’s successful implementation. The internal change agents were chosen because they had strong interpersonal and communication skills, were knowledgeable and understanding, and earned the trust and respect of the community because of their consistent interaction with the community for at least 2 years. The external facilitators of CoCoPOPP were educational outreach workers and topic experts who were external to the intervention setting and knowledgeable about their area of specialization. They met with other facilitators to provide helpful information about the evidence-based intervention and feedback when necessary.

#### Role of the facilitators

These skilled facilitators had clearly defined roles to achieve a specific objective in CoCoPOPP’s implementation and ensure consistency in the delivery process. Facilitators, especially those who interacted with community members every day, had an experience of at least 2 years in the environment of the intervention area and were fully aware of the possible challenges they were likely to face. This would allow them to be flexible, show empathy when dealing with people, and be tenacious in overcoming challenges. Thus, CoCoPOPP’s design considered high facilitation of change with input from adept internal and external facilitators.

## Discussion

Our document review and thematic analysis using the PARIHS framework showed that CoCoPOPP’s design matched a pressing health need during the COVID-19 pandemic, considered the unique context of a rural Ghanaian setting, and was guided by robust and high-quality evidence from similar interventions in past outbreaks.

The findings reveal that CoCoPOPP’s design team prioritized the effective engagement of community leaders and members. Most importantly, they went beyond community leaders to local health workers and district-level government administrators. Such engagement efforts align with Kirsch et al.’s findings on the importance of effective community involvement during global pandemics [22].

Recognizing the importance of community engagement means they factored in the role of context in the success of intervention design, as many previous studies have shown. However, they failed to acknowledge community engagement’s potential unintended negative consequences. According to Attree et al., community engagement can lead to stress and burnout for some individuals who may lose time and financial resources during community engagement activities [37]. Such adverse effects on well-being are not accounted for in any of the CoCoPOPP documents we reviewed. Therefore, future efforts to replicate the CoCoPOPP intervention must account for this challenge of potential negative consequences during community engagement exercises.

Additionally, the CoCoPOPP documents demonstrated the critical role of research during the intervention design phase. The design team engaged scientists in fields such as public health, sociology, and medicine at Yale University, Vanderbilt University, and KNUST, Ghana, to assess the quality of evidence from existing literature using tools such as GRADE and GRADE-CERQUAL (Table 4). This cross-cultural partnership aligns with existing evidence on the importance of global north-south academic partnerships for achieving social innovations [38, 39]. While we commend CoCoPOPP’s design team’s efforts to leverage academic evidence from existing literature to inform the intervention’s design, we noted that no primary data was generated from prospective feasibility studies. While we recognize the challenges of time and resource constraints in the wake of the pandemic, we also note that evidence generated from a combination of primary and secondary data would further improve an intervention’s chances of success [40, 41].

Finally, results from the thematic analysis using the PARIHS framework show that CoCoPOPP’s design team comprised a wide range of credible experts and stakeholders. These included community leaders, Cocoa360’s staff and executives, and academic partners from institutions such as Yale University. Each design team member had their roles spelt out and possessed the credibility and authenticity to ensure that the most context-dependent, evidence-driven intervention was designed. The roles of facilitation also aligned with Rifkin et al.’s framework for participation which emphasized that facilitation is not an event but a process; CoCoPOPP’s facilitators were involved in all stages of the intervention, from design to implementation and maintenance [42].

Missing in the documents was the role of power and control during facilitation. Given the differing educational and financial backgrounds between facilitators and some community members, the influence of power during the intervention design cannot be ignored [43–45]. Also, it was unclear which part of the facilitation continuum the study design focused on; whether it was just for purely one-to-one activities or intended to continuously support the intervention from design through implementation and long-term sustainability. Clarity about the intervention’s facilitation continuum would be critical in maximizing the time, skills, and energy of facilitators such as community leaders and university professors, who are usually overburdened with other responsibilities [46].

### Limitations

A significant limitation of this study is the issue of selectivity bias. Bowen argues that the chosen documents for analysis are susceptible to selectivity bias given the influence data providers, in our case, Cocoa360’s executives, can have [18]. They can give information that only aligns with a particular positive narrative they want to communicate. Additionally, document analysis of an intervention’s design is not as straightforward as this analysis may suggest. Decisions may emerge during implementation that may elicit the revision of the original design plan, a significant challenge that our analysis might not have captured. Despite these potential biases, we think the findings are still robust given the extra efforts we put into member checking, peer-debriefing, and triangulation to enhance methodological rigour [47]. Therefore, we think this study is essential to the field of implementation science, particularly for intervention design analysis in Sub-Saharan Africa, where these types of studies are limited yet are very crucial during global pandemics such as Ebola and COVID-19.

## Conclusion

CoCoPOPP’s design was consistent with the domains of the PARIHS framework since it involved the application of scientific evidence from past outbreaks, considered the unique rural context, and engaged the expertise of multiple facilitators in the community and academia. While many of the documents reviewed in this analysis indicate that Cocoa360 effectively engaged community leaders and members in CoCoPOPP’s design and implementation, it is still unclear how this would match up with primary data from interviews and questionnaires. Consequently, an important future step will be an assessment of the reach and fidelity of the CoCoPOPP intervention. Evidence from such studies will strengthen our findings from this document and framework analysis, solidifying the potential of scaling CoCoPOPP in rural communities in Ghana and beyond, particularly in other Sub-Saharan African countries with similar cultural settings.

## Data Availability

The datasets generated and/or analyzed during the current study are not publicly available due to organizational confidentiality guidelines at Cocoa360, particularly, for donor and partner reports, but are available from the corresponding author on reasonable request. Links to publicly available data have been provided in Table 1.
